# 414. Culture Clash: A Comparison of Approach to Blood Culture Diagnostic Stewardship across 3 Emergency Departments in a Healthcare Network

**DOI:** 10.1093/ofid/ofae631.128

**Published:** 2025-01-29

**Authors:** Rebecca Theophanous, Rebekah W Moehring, Erin Gettler, Timothy Plonk, Jessica Michal, Kendall Conger, Rebekah Wrenn, Jessica Seidelman

**Affiliations:** Duke University School of Medicine, Durham, North Carolina; Duke University, Durham, NC; Duke University Medical Center, Durham, NC; Duke University, Durham, NC; Duke Raleigh Hospital, Raleigh, North Carolina; Duke University, Durham, NC; Duke University, Durham, NC; Duke University School of Medicine, Durham, North Carolina

## Abstract

**Background:**

Blood culture (BCx) diagnostic stewardship aids in accurately identifying microbial pathogens and guiding appropriate antibiotic therapy. We compared effects of a BCx algorithm (Fig 1) on BCx rates (BCx/100 ED visits) in the emergency department (ED) using two implementation approaches: 1) an intensive approach including weekly, individualized BCx appropriateness data feedback and modifications to electronic health record order sets, and 2) a passive approach with group-level, BCx appropriateness data feedback of a random BCx sample.

**Figure 1:**
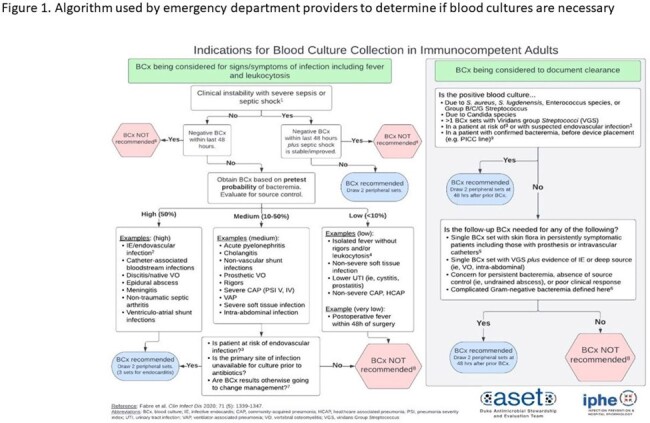
ED Blood culture algorithm Algorithm used by emergency department providers to determine if blood cultures are necessary

**Methods:**

We performed a quasi-experimental pre-/post-intervention study from 12/1/2020 to 2/29/2024 using data from 3 academic-affiliated EDs. The BCx algorithm was introduced in 12/2022 in ED1. 7 ED providers reviewed BCxs ordered each week for appropriateness of BCx indication according to the algorithm and provided weekly, individualized feedback to colleagues regarding their performance in. In addition, BCx were removed from order sets for low-risk clinical scenarios, such as simple cystitis or community-acquired pneumonia. The algorithm was introduced to ED2 in 7/2023. In ED2, a pharmacist reviewed 5 BCx per week for appropriateness and one ED physician provided a secondary review when the clinical scenario was unclear. Feedback was provided to ED leadership every 2 months regarding appropriateness and BCx utilization. ED3 served as control and did not have the algorithm introduced. BCx rates were analyzed using interrupted time series models evaluating each ED separately. Incidence rate ratios (IRR) compared BCx rates before the intervention to BCx rates after the intervention.

**Figure 2.**
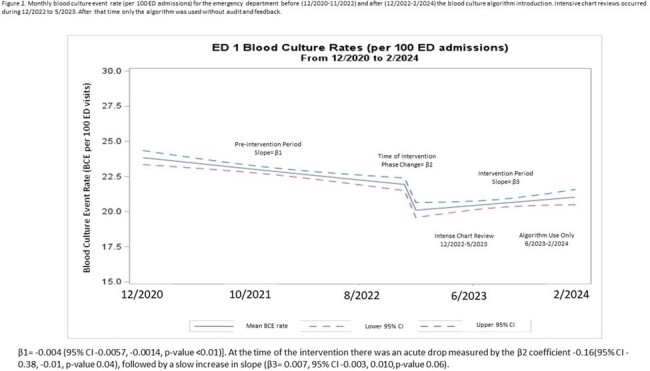
Monthly blood culture event rate (per 100 ED admissions) for the emergency department before (12/2020-11/2022) and after (12/2022-2/2024) the blood culture algorithm introduction. Intensive chart reviews occurred during 12/2022 to 5/2023. After that time only the algorithm was used without audit and feedback. β1= -0.004 (95% CI -0.0057, -0.0014, p-value <0.01)]. At the time of the intervention there was an acute drop measured by the β2 coefficient -0.16(95% CI -0.38, -0.01, p-value 0.04), followed by a slow increase in slope (β3= 0.007, 95% CI -0.003, 0.010,p-value 0.06).

**Results:**

A total of 105,975 BCxs over 572,776 ED visits were included in the analysis. ED1 saw a 25% decrease in BCx rate with IRR 0.80 (95% CI 0.74, 0.86, p-value 0.01). (Fig 2) ED2 experienced a 4% decrease in BCx rate with IRR 0.72 (95% CI 0.54, 0.95, p-value 0.02). (Fig 3) ED3 did not experience any change (Fig 4).

**Figure 3.**
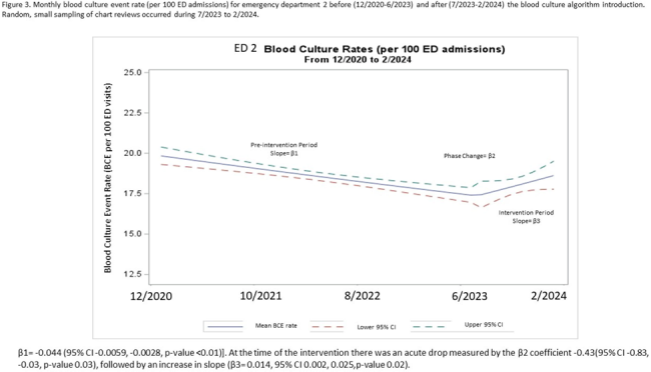
Monthly blood culture event rate (per 100 ED admissions) for emergency department 2 before (12/2020-6/2023) and after (7/2023-2/2024) the blood culture algorithm introduction. Random, small sampling of chart reviews occurred during 7/2023 to 2/2024. β1= -0.044 (95% CI -0.0059, -0.0028, p-value <0.01)]. At the time of the intervention there was an acute drop measured by the β2 coefficient -0.43(95% CI -0.83, -0.03, p-value 0.03), followed by an increase in slope (β3= 0.014, 95% CI 0.002, 0.025,p-value 0.02).

**Conclusion:**

The BCx rate was most significantly impacted in ED1 by combining audit and feedback with modifications to the electronic medical record. However, both EDs saw drift back towards prior BCx utilization rates after the intervention. Sustainability can likely be improved by implementing computerized clinical decision support systems.

**Figure 4.**
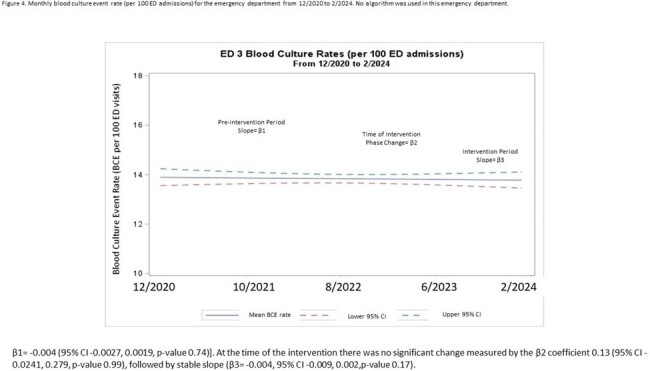
Monthly blood culture event rate (per 100 ED admissions) for the emergency department from 12/2020 to 2/2024. No algorithm was used in this emergency department. β1= -0.004 (95% CI -0.0027, 0.0019, p-value 0.74)]. At the time of the intervention there was no significant change measured by the β2 coefficient 0.13 (95% CI -0.0241, 0.279, p-value 0.99), followed by stable slope (β3= -0.004, 95% CI -0.009, 0.002,p-value 0.17).

**Disclosures:**

**Rebekah W. Moehring, MD, MPH, FIDSA, FSHEA**, UpToDate, Inc.: Author Royalties **Jessica Seidelman, MD, MPH**, 3M: Expert Testimony

